# Anti-*Aspergillus* Activities of the Respiratory Epithelium in Health and Disease

**DOI:** 10.3390/jof4010008

**Published:** 2018-01-08

**Authors:** Margherita Bertuzzi, Gemma E. Hayes, Uju J. Icheoku, Norman van Rhijn, David W. Denning, Nir Osherov, Elaine M. Bignell

**Affiliations:** 1 Manchester Fungal Infection Group, Faculty of Biology, Medicine and Health, University of Manchester, Manchester M13 9NT, UK; margherita.bertuzzi@manchester.ac.uk (M.B.); pharmjoyce@gmail.com (U.J.I.); norman.vanrhijn@postgrad.manchester.ac.uk (N.v.R.); 2 Northern Devon Healthcare NHS Trust, North Devon District Hospital, Raleigh Park, Barnstaple EX31 4JB, UK; gemmahayes1@nhs.net; 3 The National Aspergillosis Centre, Education and Research Centre, Wythenshawe Hospital, Manchester University NHS Foundation Trust, Manchester M23 9LT, UK; ddenning@manchester.ac.uk; 4 Department of Clinical Microbiology and Immunology, Sackler School of Medicine, Tel Aviv University, Tel Aviv 69978, Israel; nosherov@post.tau.ac.il

**Keywords:** *Aspergillus fumigatus*, respiratory epithelium, airway epithelial cells (AECs), spore uptake, epithelial responses, morphotypes, fungal pathogenesis, internalization

## Abstract

Respiratory epithelia fulfil multiple roles beyond that of gaseous exchange, also acting as primary custodians of lung sterility and inflammatory homeostasis. Inhaled fungal spores pose a continual antigenic, and potentially pathogenic, challenge to lung integrity against which the human respiratory mucosa has developed various tolerance and defence strategies. However, respiratory disease and immune dysfunction frequently render the human lung susceptible to fungal diseases, the most common of which are the aspergilloses, a group of syndromes caused by inhaled spores of *Aspergillus fumigatus*. Inhaled *Aspergillus* spores enter into a multiplicity of interactions with respiratory epithelia, the mechanistic bases of which are only just becoming recognized as important drivers of disease, as well as possible therapeutic targets. In this mini-review we examine current understanding of *Aspergillus*-epithelial interactions and, based upon the very latest developments in the field, we explore two apparently opposing schools of thought which view epithelial uptake of *Aspergillus* spores as either a curative or disease-exacerbating event.

## 1. Introduction

*Aspergillus*-related diseases occur frequently in the human population and pathological outcomes are diverse [1]. Amongst more than 2.3 million episodes occurring annually in Europe alone, the vast majority of aspergilloses initiate in the respiratory tract via fungal spore inhalation resulting in up to 2 million cases of allergic disease in asthma and cystic fibrosis sufferers, and more than 300,000 cases of semi-invasive or invasive aspergillosis of which 70,000 prove fatal [2]. A unifying feature of all respiratory aspergilloses is the interaction of inhaled fungal particles with the respiratory epithelium, the outcome of which differs dramatically according to the respiratory niche(s) involved and the immunological status of the affected host.

A detailed understanding of the host and pathogen processes driving healthy clearance of fungal spores, as well as the mechanistic defects which drive adverse pathologies will better enable a more targeted therapeutic strategy in the future. To this end we review the most recent developments in the field, with a particular focus upon spore attachment to, and uptake by the respiratory epithelium, epithelial responses to *A. fumigatus* challenge, and their likely impacts upon disease outcomes. As authoritative reviews of the earlier literature have already dealt with experimental systems, founding concepts in the field, and relevant pathogenicity factors [3,4,5], we restrict our focus to aspects of the host-pathogen interaction having most pertinence to the outcome of *Aspergillus*-related lung diseases.

It is now widely accepted that epithelial uptake of *A. fumigatus* spores is an important component of the interaction with cultured human epithelia leading either to fungal killing or intraphagosomal occupation of airway epithelial cells (AECs). Despite multiple studies reporting spore internalisation by immortalised and/or primary AECs in in vitro infection systems and ex vivo organ culture models [6,7,8,9,10,11,12,13,14], no compelling published evidence of in vivo spore internalisation by AECs is available. On the contrary, using a novel bioimaging approach combined with transmission electron microscopy (TEM), Rammaert et al. recently demonstrated the absence, in vivo, of fungal spore internalisation in the bronchial epithelium of mice [15]. Due to the resolution limit of TEM, and the likely low frequency of spore uptake in vivo, these findings do not exclude the possibility that spore internalisation might occur during mammalian infection. Furthermore, the observation of Rammaert et al. derives from the study of bronchial, but not alveolar, epithelium [15]. The relevance of spore uptake to health likely encompasses several outcomes of the host-pathogen interaction, including those where uptake serves as a useful means of neutralising low-level spore exposure or as driving invasive growth and pathogenesis [6,8,14,16,17] where fungal killing is unachievable (such as during disease [14]). Here, we examine the latest advances in understanding of the host-*A. fumigatus* interaction and its role in driving or curtailing lung disease.

**Figure 1:**
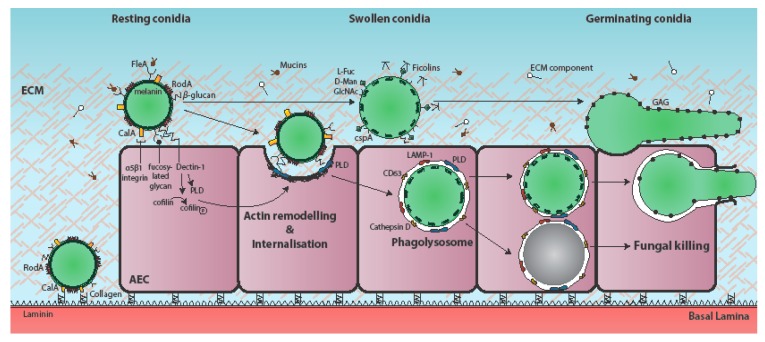
Temporal and mechanistic basis of *A. fumigatus* attachment to, and uptake by, the respiratory epithelium. Respiratory aspergilloses commence with interaction of inhaled fungal particles with the respiratory epithelium. During vegetative growth of the pathogen, individual airway epithelial cells (AECs) may become iteratively exposed to one or multiple fungal morphotypes, as well as cell surface-associated and secreted fungal factors [8]. *A. fumigatus* conidia bind to AECs, extracellular matrix (ECM) components, and basement membrane components [21,22,23]. The immunoprotective conidial hydrophobin RodA is required for adherence to collagen [10,18,19,20,24] and the fucose-specific conidial lectin FleA mediates fucose-dependent binding of *A. fumigatus* conidia to airway mucin [25,26]. The cell surface protein CspA, which becomes unmasked during conidial germination, is also required for adhesion to A549-derived ECM [18]. The H-ficolin opsonin binds to *A. fumigatus* conidia via l-fucose, d-mannose and *N*-acetylglucosamine (GalNAc) on the conidial surface and moderates adhesion to A549 cells [27,28,29]. The secreted and hyphal cell wall-associated exopolysaccharide galactosaminogalactan (GAG) mediates adherence of *A. fumigatus* hyphae to fibronectin and epithelial cells [19]. CalA is the first identified invasin of *A. fumigatus* and is required for epithelial entry in a α5β1 integrin-dependent manner [17]. Spore internalisation is thought to be dependent on E-cadherin [30,31,32], and the actin regulators phospholipase D (PLD) [9] and cofilin-1 [33]. PLD co-localises with internalised conidia, as do the late endosomal/lysosomal markers LAMP-1, CD63, and cathepsin D [6,9]. Most internalised conidia are killed, but a few remain viable and eventually germinate to escape the phagolysosome without lysis of the host cell [6]. Induction of PLD following exposure to β-1,3-glucan on the surface of germinating conidia has been demonstrated to occur in a Dectin-1-dependent manner [9]. Conidial dihydroxynaphthalane (DHN) melanin increases internalisation of *A. fumigatus* spores by A549 cells and by preventing the acidification of AEC phagolysosomes, promotes spore viability [34].

**Figure 2:**
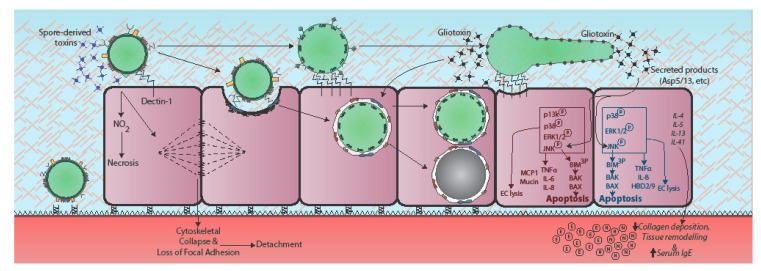
Epithelial responses to *A. fumigatus.* Epithelial responses to *A. fumigatus* vary dramatically according to fungal morphotype and host cell origin, the latter denoted in red for alveolar epithelial cells and blue for bronchial epithelial cells. Contact with *A. fumigatus* spores prompts rapid cytoskeletal reorganisation and loss of focal adherence, possibly via spore-associated toxins [35]. In bronchial epithelial cells recognition of germinating conidia and hyphae leads to upregulation of Dectin-1 expression at the host cell membrane and phosphorylation-mediated activation of phosphatidylinositol3-kinase (PI3K), mitogen activated protein kinase (MAPK) p38 and ERK1/2 signalling, leading to TNFα, IL-8, and β-defensin expression (HBD-2 and -9) [36,37,38]. In AECs, challenge with *A. fumigatus* culture filtrate prompts MAPK ERK1/2, p38, and c-Jun N-terminal protein kinases (JNK) phosphorylation, in a partially fungal protease-dependent manner, leading to lytic death of the host cell [39,40]. JNK or p38 inhibitors protect against host damage [39]. The secreted products of cultured *A. fumigatus* also elicit remodelling of the respiratory epithelium involving heightened expression of classical Th2 cytokines (IL-4, IL-5, IL-13), serum IgE, collagen deposition, and neutrophil (N) and eosinophil (E) recruitment [41]. Gliotoxin exposure induces cytoskeletal collapse, cell peeling, and apoptosis. Gt activates the JNK pathway leading to apoptosis. JNK triple-phosphorylates the pro-apoptotic protein Bim, leading to pro-apoptotic Bak and Bax activation, mitochondrial membrane permeabilization, and apoptosis [42,43,44,45]. Italicised text indicates responses observed in whole animals or humans.

Due to the occurrence of an obligatory morphological shift during conidial germination of *Aspergillus* species, *Aspergillus*-epithelial interactions are most usefully regarded within a temporal framework commencing with spore-mediated host interactions, and outcomes thereof, and ending with either fungal death, or fungal invasion of the lung parenchyma (Figure 1 and Figure 2). Germination of inhaled *Aspergillus* spores involves a period of isotropic growth (swelling) which, if not apprehended, will be followed by the outgrowth of a tip-extending elongated cell called a primary hypha, which actively secretes fungal proteases and secondary metabolites whilst growing in a polarised fashion into surrounding tissues [8]. Since the cell surface of resting *A. fumigatus* conidia differs markedly from that of metabolically active, germinating spores and hyphae [18,19,20], and cytotoxic secreted factors derive predominantly from mature hyphal cells [8], it is likely that within the time frame of the host-pathogen interaction, individual epithelial cells will become iteratively exposed to one or multiple fungal morphotypes, as well as fungal cell surface-associated and secreted factors. Host responses to *Aspergillus* encounters, including the ability to contain the pathogenic threat and the nature of ensuing host damage, vary according to both the fungal and epithelial cell types involved (Figure 2), and are critically impacted by host immune status.

## 2. Attachment of *A. fumigatus* to the Respiratory Epithelium

*A. fumigatus* conidia bind in a concentration-dependent manner to A549 epithelia and extracellular matrix (ECM), and to purified basement membrane components, such as fibrinogen, fibronectin, laminin, type I collagen, and type IV collagen [21,22,23]. Basement membrane components are not normally accessible to pathogens in healthy lungs, but often become exposed in lungs of asthmatic patients, hence, adhesion to the basal lamina could facilitate fungal infection and persistence, exacerbating the risk of aspergillosis in this cohort of patients [21], as well as in other disease settings where integrity of the pulmonary epithelium is diminished.

Several fungal surface components, many of which are expressed in a morphotype-specific manner, play a role in *A. fumigatus* attachment to AECs [18,19,20], thereby reflecting the structural complexity and dynamism of the cell wall during germination and hyphal growth. For example, the conidial hydrophobin RodA, which renders *A. fumigatus* spores immunologically inert, but is shed from the spore surface during germination, is required for adherence to collagen, but not to A549 cells or other basement membrane components, such as laminin and fibronectin [24] (Figure 1). In the context of healthy defence against inhaled fungal spores, RodA is argued as being a facilitator of immune evasion at the earliest of time points following spore inhalation [46]. Since RodA extracted from *A. fumigatus* conidia is immunologically inert and fails to induce human dendritic cell maturation, it might have a role to play in inducing human tolerance to everyday spore exposure [46]. However, in diseased settings, and on the basis of current evidence, any role for RodA in the establishment of epithelial colonisation would necessarily involve (a) ungerminated *A. fumigatus* spores and (b) a pathological setting in which collagen is exposed.

Morphotype-specific adhesion factors are also expressed on swollen and germinating spores and hyphae, for example, CspA [18], which becomes unmasked during conidial germination [47], and is required for full adhesion to A549-derived ECM (Figure 1). Notably, a *cspA* null mutant exhibits only a 50% reduction in ECM binding relative to the progenitor clinical isolate, thereby supporting the hypothesis of multiple conidial adhesion factors expressed by *A. fumigatus* [47]. Furthermore, a recent study of 30 clinical and environmental isolates screened for conidial phenotypes and adherence to A549 cells, revealed four *A. fumigatus* strains with decreased adherence to A549 cells having a reduced conidial size, number, or hydrophobicity [20]. RNAseq analysis of the four most highly adherent isolates revealed a common cluster of 31 differentially-expressed genes during conidial formation, two of which (AFUA_4G01030 and AFUA_4G08805) were found by mutational analysis to be important for adhesion to AECs [20].

Due to the abundance of fucose on human cells, the recent discovery of an *A. fumigatus* soluble fucose-specific lectin, FleA [25,26], prompted speculation that FleA might be the conidial surface protein responsible for directing attachment of *A. fumigatus* to host cells, such as epithelial cells and macrophages, via membrane-tethered mucins [48]. Recombinant FleA prompts haemagglutination of human red blood cells in a fucose-dependent manner, directly binds fucose-containing oligosaccharides in glycan array studies and elicits IL-8 expression in cultured BEAS-2B bronchial epithelial cells. An anti-FleA rabbit polyclonal antibody revealed conidia-, but not hypha-, specific localisation of FleA in microscopy-mediated immunofluorescence assays [48]. Subsequent analyses of FleA-mediated glycoprotein binding revealed that FleA mediates binding of *A. fumigatus* conidia to airway mucin in a fucose-dependent manner (Figure 1) and is a necessary prerequisite for macrophage-mediated phagocytosis of *A. fumigatus* spores. Concordant with a role for FleA in driving host-mediated clearance of inhaled *A. fumigatus* spores, a *fleA* null mutant promoted invasive growth and heightened inflammatory cell recruitment in immunocompetent mice, however, the role of FleA in driving epithelial uptake of *A. fumigatus* spores remains untested [48].

Carbohydrate residues on the surface of *A. fumigatus* germinating conidia are ligands for ficolins (M-, L-, and H-ficolin in human serum), a family of lectin-like opsonins whose secretion is increased in bronchoalveolar lavage fluid and infected lungs of individuals with invasive aspergillosis [27,28,29] (Figure 1). Competitive binding assays using a range of carbohydrates and H-ficolin pre-incubation revealed that H-ficolin binds to *A. fumigatus* conidia via l-fucose, d-mannose, and *N*-acetylglucosamine (GalNAc) on the conidial surface and in a calcium- and pH-dependent manner, with maximal binding observed in acidic conditions (pH 5.7). At this pH, H-ficolin opsonised conidia are twice as adherent to A549 cells relative to their non-opsonised counterparts [29]. Recombinant M-ficolin was found via glycan arrays [49] to bind to acetylated compounds including GalNAc, and via pull-down assays [27] to bind to chitin and β-1,3 glucan on the *A. fumigatus* cell wall. Quantitation of C4 complement consumption and deposition and IL-8 ELISA demonstrated that opsonisation of the alkali-insoluble fraction of *A. fumigatus* cell wall with recombinant M-ficolin mediates complement activation and potentiates IL-8 secretion by A549 cells in a dose-dependent manner. Although H-ficolin has been demonstrated to positively influence binding of *A. fumigatus* spores to A549 cells [29], the role of M-ficolin adherence to host cells remains unexplored. In support of a protective role for ficolin-mediated activities during *A. fumigatus* infection, a recent study showed that mice lacking both ficolin A and B (murine orthologues of the human L- and M-ficolins [50]) suffer significantly higher pulmonary fungal burden compared to their wild-type counterparts [51]. Cytokine ELISA of broncoalveolar lavage fluids (BALF) revealed a trend towards decreased production of IL-1β, IL-6, and TNFα in the lungs of *A. fumigatus*-infected ficolin-deficient mice [51]. However, defective fungal clearance did not correlate with muted recruitment of inflammatory cells or aberrant complement activation during *A. fumigatus* infection as measured via quantification of myeloperoxidase in murine lung homogenates and complement component C3a in BALFs.

E-cadherin has also been suggested to play a role in *A. fumigatus* spore adhesion to, and uptake by, A549 cells given the reduction in E-cadherin expression via application of a blocking antibody or siRNA expression results in reduced cell-conidial association and conidial phagocytosis [31]. Accordingly, co-immunoprecipitation and mass spectrometry analysis following co-incubation of *A. fumigatus* spore lysates with purified recombinant human E-cadherin recently identified the metalloprotease designated as MEP (or AFUA_8G07080), and another two uncharacterised proteins, AFUA_1G11480 and AFUA_6G02870, as putative mediators of *A. fumigatus* spore binding to the respiratory epithelium via the adhesion molecule E-cadherin although this hypothesis remains to be substantiated [30,31,32].

The secreted and hyphal cell wall-associated exopolysaccharide galactosaminogalactan (GAG) has been demonstrated to mediate a number of virulence-associated traits, including adherence of *A. fumigatus* hyphae to fibronectin and A549 epithelial cells [19] (Figure 1). GAG is a heteroglycan composed of galactose and *N*-acetyl-galactosamine (GalNAc), produced by hyphae but not conidia [52], via the activity of the fungal epimerase Uge3. Uge3 mediates the synthesis of the GalNAc component of GAG [19,53] and a *uge3-*deficient mutant shows a 10-fold reduction in adhesion to A549 monolayers compared to the respective parental isolate [19]. Uge3 is also required for the induction of epithelial cell damage by *A. fumigatus*, as a *uge3-*deficient mutant is 10-fold less able to cause lysis of A549 cells after 16 h of infection compared to its wild-type counterpart [19]. Furthermore, survival analyses and fungal burden analyses performed in cortisone acetate-treated or leukopenic mice indicate that the Δ*uge3* mutant is less efficient at colonising the murine lung [19], suggesting an important role for adherence to host cells during infection. Recently, the deacetylation of GalNAc residues within GAG has been identified as an essential requirement for the adherence of GAG to hyphae. The Agd3 deacetylase is required for the deacetylation of GAG [54], and a Δ*agd3* isolate does not display detectable cell wall-associated GAG as measured by direct immunofluorescence with the GalNAc-specific soybean agglutinin lectin [54]. More importantly, a Δ*agd3* mutant is unable to adhere to negatively charged surfaces because de-*N*-acetylated GAG confers a positive charge on the hyphal surface [54]. Deletion of *agd3* attenuates *A. fumigatus* virulence in a leukopenic mouse model of invasive aspergillosis, demonstrating that deacetylation of GAG is required for full virulence of *A. fumigatus* [54]. The epithelial receptor for GAG is still unknown.

## 3. Epithelial Uptake of *A. fumigatus* Spores

Cultured bronchial cells and type II alveolar cells are able to internalise approximately 30–50% of adherent *A. fumigatus* conidia in a concentration- and time-dependent manner [6,7,8,9,12,13,14,32,33,55]. The fungal ligands required for uptake of *A. fumigatus* by AECs remain largely unknown but, recently, a thaumatin-like protein, namely CalA, was identified as the first invasin of *A. fumigatus* [17]. Analogously to a previously identified *A. nidulans calA* gene homologue, which is highly expressed during and required for, spore germination and hyphal formation [56], an *A. fumigatus* Δ*calA* mutant exhibits delayed germination and curved hyphal tips when grown in tissue-culture medium. *A. fumigatus* CalA was predicted as a candidate adhesin via bioinformatics analysis, supported by the finding that a recombinant version of CalA produced in *Escherichia coli* is able to bind laminin and AECs in vitro [57]. However, an *A. fumigatus* strain lacking *calA* was not deficient in binding immobilized laminin or A549 cells, indicating that any role CalA plays in adhesion can be served by other adhesion factors [17]. A differential fluorescence approach demonstrated that, compared to the respective parental isolate, a *calA* null mutant shows a significant reduction (50%) in uptake by A549 cells after 2.5 h of infection. CalA therefore mediates, at least in part, fungal internalisation by A549 and primary human alveolar epithelial cells. Both whole-cell affinity purification, and subsequent immunoblotting with anti-integrin antibodies, indicated that CalA-dependent internalisation is mediated by direct interaction with the host cell receptor α5β1 integrin [17] (Figure 1). Accordingly, in differential fluorescence assays, both β1 and α5 integrin antibody- and siRNA-mediated inhibition reduce internalisation of *A. fumigatus* by ~50% when infecting A549 monolayers for 2.5 h [17]. Importantly, CalA is also required for normal virulence and lung invasion as demonstrated via survival and fungal burden analyses in cortisone- acetate treated mice following aerosol infection with *A. fumigatus* conidia. In the same infection model an anti-CalA antibody reduced *A. fumigatus* uptake by A549 cells by 50% and, when injected intraperitoneally prior to *A. fumigatus* infection, increased the survival of the infected mice by 20%, suggesting CalA to be a plausible target for novel therapeutic interventions.

Redundancy of function apparently also extends to the host cell repertoire of internalisation factors. Spore internalisation is dependent on two crucial regulators of host actin dynamics, phospholipase D (PLD) and cofilin-1, whose activation and phosphorylation state, respectively, are modulated by exposure to β-1,3-glucan on the surface of germinating conidia [9,33] (Figure 1). Accordingly, chemical or siRNA-mediated downregulation of host PLD or aberrant phosphorylation of cofilin-1 via the RhoA-ROCK-LIM kinase pathway causes a decrease in *A. fumigatus* internalisation [9,33]. In A549 cells, PLD co-localises with internalised conidia [9], which also quickly become co-localised with late endosomal/lysosomal markers, such as LAMP-1, and CD63 and cathepsin D, indicating rapid trafficking through the endosomal system to the phagolysosomes [6]. Following phagolysosomal acidification most of the internalised conidia are killed, but after 24 h 3% of the internalised conidia remain viable and 34% of these can eventually germinate to escape the phagolysosome by 36 h, without lysis of the host cell [6]. Two additional host proteins (ARC and EGR1) upregulated at 8 h in response to *A. fumigatus* challenge, have been implicated as influencing uptake of conidia by A549 cells, as siRNA mediated knockdown of the respective gene functions diminished conidial uptake by approximately 20% and 40%, respectively [58].

In AEC, induction of PLD following exposure to β-1,3-glucan on the surface of germinating conidia has been demonstrated to occur in a Dectin-1 dependent manner [9]. Accordingly, anti-Dectin-1 antibodies significantly reduce *A. fumigatus* conidia internalisation by A549 cells [8,9]. Supporting a role for Dectin-1 in mediating curative spore clearance in vivo a high risk of invasive pulmonary aspergillosis has been reported in hematopoietic stem cell transplantees carrying a non-functional truncated version of Dectin-1 [59]. This suggests that Dectin-1 mediated activities of non-hematopoietic cells, such as AECs, play a crucial role in defence against aspergillosis. Studies in A549 and immortalised human bronchial epithelial cells (HBECs) have revealed conflicting reports [9,36,60,61] of Dectin-1 expression levels whereby immunohistochemical staining of human lung sections reveals predominantly apical expression of Dectin-1 on bronchial and alveolar human epithelium [60], while flow cytometric analyses of primary small airway epithelial cells (SAECs) and primary HBECs confirmed Dectin-1 expression [60], but failed to detect the receptor in the A549 cell line unless induced by challenge with *Mycobacterium tuberculosis* or *A. fumigatus* [61]. Given that Han et al. (2011) reported constitutive Dectin-1 expression in A549 cells [9], Sun et al., reported Dectin-1 expression in a papilloma virus-immortalised human bronchial epithelial cell line [47] and anti-Dectin-1 antibodies significantly ameliorate conidial internalisation and epithelial damage following *A. fumigatus* infection [8], it is feasible that Dectin-1 is specifically responsive to *A. fumigatus* challenge in both cultured and primary respiratory epithelia. A targeted comparative study will be necessary in order to resolve this important question.

Conidial dihydroxynaphthalane (DHN) melanin increases internalisation of *A. fumigatus* spores by A549 cells [34], as demonstrated by an analysis of epithelial uptake of non-pigmented Δ*pksP* mutants versus that of the respective parental isolate. In professional phagocytes DHN melanin on the surface of *A. fumigatus* spores prevents the acidification of phagolysosomes and, consequently, spore killing by professional phagocytes [62,63,64]. Concordant with intraphagosomal behaviour in macrophages, melanised wild-type *A. fumigatus* spores inhibit the acidification of A549 phagolysosomes thereby achieving higher intracellular survival rates compared to those of the strain Δ*pksP* [34]. In human monocyte-derived macrophages, intracellular swelling of *A. fumigatus* conidia and, in particular, cell wall melanin removal during germination, is required for the activation of a specialized autophagy pathway called LC3-associated phagocytosis (LAP) which ultimately results in fungal killing [65,66]. Accordingly, a non-pigmented Δ*pksP* mutant lacking DHN-melanin is avirulent in wild-type mice [17,56,57], but not in mice with conditional inactivation of *Atg5* in hematopoietic cells. The role of autophagy in fungal killing within AECs remains untested, as does the mechanistic basis of AEC-mediated *A. fumigatus* killing.

## 4. Epithelial Responses to *A. fumigatus*

In common with the direct physical interactions between host and pathogen described above, it is now clear that epithelial responses to *A. fumigatus* also vary dramatically according to the fungal morphotype and host cell origin. Interpretation of findings in this field is therefore significantly confounded by immense experimental heterogeneity, including multiplicity of infection, fungal isolate, culture medium, etc. For example, in cultured HBECs (BEAS-2B), recognition of germinating (but not resting) conidia and hyphae leads to activation of phosphatidylinositol3-kinase (PI3K), mitogen-activated protein kinase (MAPK) p38 and ERK1/2 after 10 h of co-incubation with *A. fumigatus* conidia [37]. Similarly, expression and production of β-defensin 2 (HBD-2) and HBD-9 is significantly increased in response to live, swollen conidia, but not in response to resting conidia or heat dried hyphal fragments [38]. Further, when challenged with *A. fumigatus* conidia a papilloma virus-immortalised bronchial epithelial cell line upregulates Dectin-1 expression at the cell membrane at 6 and 18 h post challenge with resting conidia compared to challenge with heat-killed conidia, latex beads, or laminarin, thereby suggesting that fungal viability, rather than mere particulate contact with epithelial cells is a critical factor in driving Dectin-1 mediated epithelial activities [36]. Other groups have reported that physical contact with conidia, irrespective of fungal viability, is also important [67], as only inactivated resting, but not swollen or germinated conidia, induced IFN-β signalling through the RIP-1/TBK1 pathway in human primary bronchial epithelial cells. It is well documented that mere contact with *A. fumigatus* spores is sufficient to prompt actin cytoskeletal reorganisation and loss of focal adherence [35], however, the mechanistic basis of such cytotoxicity remains unknown. Recently, Gauthier et al., identified five main spore-associated toxins: tryptoquivaline F, fumiquinazoline C, questin, monomethylsulochrin, and trypacidin, amongst which trypacidin was shown to be the most toxic, as evidenced by MT-mediated cytotoxicity assays and LDH quantitation in A549 cells and primary HBECs [68]. In A549 epithelial cells, lysis was detectable as early as 1 h post exposure and was directly proportional to the dose of trypacidin used [68]. Trypacidin triggers cell death by initiating intracellular formation of nitric oxide, leading to death of the cells after about 24 h by necrosis. It is as yet unclear whether these toxins are retained in conidia killed by various methods.

Concordant with a role for mature hyphal morphotypes in prompting host responses, fluorescence activated cell sorting (FACS) analysis and confocal immunofluorescence microscopy, applied to papilloma virus-immortalised HBECs, revealed a time-dependent increase in Dectin-1 cell surface expression peaking at 24 h post-infection and correlating with transcriptional upregulation of TNFα, IL-8, HBD-2, and HBD-9, an effect which could be abrogated by pre-incubation with an anti-Dectin-1 antibody [36]. Accordingly, in a leukopenic murine model of invasive pulmonary aspergillosis, expression of TNFα, GM-CSF, IL-1β, and IL-10, and secretion of TNFα and GM-CSF, was significantly increased following adenovirus mediated Dectin-1 upregulation in lung tissue resulting in increased neutrophil recruitment into the lungs, lower fungal burden, and a higher survival rate in response to intratracheal infection with 3 × 10^5^ *A. fumigatus* conidia [69]. 

Proteomic analysis of the secretome of BEAS-2B cells infected with *A. fumigatus* via difference in-gel electrophoresis (DIGE) and immunoblotting showed a time-dependent induction of the release of lysosomal enzymes (namely NAGase, cathepsin B, and cathepsin D) and proteins of the thioredoxin system in response to fungal challenge [70]. Cell degranulation required live fungus, whereby heat-killed spores or hyphae were unable to induce NAGase release [70]. Furthermore, PI3K and the p38 MAPK inhibitors diminished NAGase release by approximately 60% and 70%, respectively, thereby demonstrating that lysosomal enzyme release is partly mediated by PI3K and the p38 MAPK pathways [70]. The demonstration that *A. fumigatus* induces the release and activation of lysosomal enzymes by bronchial cells and proteins of the redox system, suggests that degranulation of lysosomal enzymes may participate in controlling fungal growth and human cell self-damage [70].

Differential responses to *A. fumigatus* conidia, germ tubes, and secreted fungal products are also mounted by cultured alveolar A549 cells. Exposure of A549 cells to *A. fumigatus* conidia for 3 h resulted in only a slight increase in ERK1/2 phosphorylation compared to polystyrene beads and no significant MAPK p38 activation, as monitored by Western blot using anti-p-ERK and anti-p-p38 specific antibodies [39]. In contrast, challenge with culture filtrate from *A. fumigatus* at the same time point led to MAPK ERK1/2, p38, and JNK phosphorylation. ERK1/2 and JNK, but not p38 phosphorylation, was abolished during exposure to culture filtrate from an *A. fumigatus* mutant (Δ*prtT*) lacking the PrtT transcription factor suggesting a role for proteolytic activities of secreted fungal proteases in prompting host responses [39]. Recent RNAseq analyses of A549 gene expression at 8 h post-infection with viable *A. fumigatus* conidia revealed upregulation of as many as 302 genes compared to uninfected cells most of which are involved in immune response, chemotaxis, cell activation, and regulation of phosphorylation [58]. Analysis of the mRNA levels of inflammatory cytokines by targeted qRT-PCR showed significant increase in expression of IL-6, IL-8, and TNFα from 6–8 h post interaction with conidia [58].

Given the wide range of host cell types, fungal challenges, and time-points employed for studying *A. fumigatus*-respiratory epithelial interactions it has not yet been possible to assemble a coherent account of the host-pathogen interaction [3,4,37,38,39,58,67,71]. The iterative nature of host contact with different fungal morphotypes as fungal growth progresses suggests that localised manipulation of host responses and host damage elicited by *A. fumigatus* challenge occurs in a stepwise and cumulative fashion. In support of this hypothesis, the recent discovery of a tissue non-invasive *A. fumigatus* mutant lacking the pH-responsive transcription factor PacC [8] revealed stage-specific features of the *A. fumigatus*-A549 interaction to comprise of a minimum of two mechanistically distinct phases of damage, the first (prior to 16 h of co-incubation) occurring in a contact-mediated manner and independently of secreted fungal proteins and the second occurring via the action of secreted fungal products. PacC null mutants were found to exhibit a non-invasive phenotype in A549 alveolar epithelia, where contact-mediated detachment and epithelial decay elicited by fungal culture filtrate were highly attenuated relative to infection with non-mutated clinical isolates. This phenotype was replicated in leukopenic mice where Δ*pacC* conidia germinate but fail to invade the respiratory epithelium [8].

For cultured A549 epithelia Icheoku et al. recently constructed the first comprehensive temporal map of host responses to *A. fumigatus* revealing discrete responses to resting, swollen, and germinating spores, hyphae, and secreted fungal products [40]. Modest (two-fold relative to PBS challenge), but sustained, phosphorylation of IκBα was observed from as early as 4 h post-infection and was maintained throughout a 48 h time-course with viable *A. fumigatus*. In stark contrast, however, hyphal maturation or direct exposure to culture filtrates from mature (48 h) fungal cultures reproducibly elicited heightened phosphorylation of JNK (seven-fold), p38 (six-fold), and ERK1/2 (two-fold) relative to PBS challenge. Epithelial cell lysis, quantified via a lactate dehydrogenase enzyme assay, was first measurable at 12 h post-infection and coincided with initiation of hyphal growth indicating significant toxicity of secreted fungal products. The application of JNK or p38 inhibitors prior to fungal challenge reduced lytic cell death by 70% and 40%, respectively, indicating that the host response to secreted fungal products is an important driver of host damage. The latter finding is concordant with that of Sharon et al. [39], whereby protease inhibitors were found to ameliorate epithelial damage caused by fungal culture filtrate [39]. The same authors also demonstrated that inhibition of JNK or ERK1/2 kinase activity substantially decreased CF-induced cell damage, including cell peeling, actin-cytoskeleton damage, and reduction in metabolic activity and necrotic death. In NCI-H292 bronchial epithelial cells, increased production of IL-6, IL-8, MCP-1, and mucin in response to *A. fumigatus* culture filtrate for 48 h is also abrogated by treating with serine protease inhibitors [71] (Figure 2).

Due to the difficulties in isolating primary alveolar epithelial cells (AECs) from human patients, animal models remain, notwithstanding the significant influence of host cell isolation and handling on cellular physiology, the closest alternative for the study of host-*A. fumigatus* interactions. Seddigh et al. recently developed a novel negative immunomagnetic protocol for isolating untouched primary AECs from murine lung for quantitative-label free in vivo proteomics [72]. By circumventing flow-cytometry-based cell sorting, which, by itself, massively influences cellular physiology, this approach permitted the targeted comparative ex vivo analysis, in nine biological replicates, of type II AECs from *A. fumigatus*-infected and sham-infected C57BL/6 mice at 24 h post-challenge. The study demonstrated a remarkable increase in abundance of L-amino acid oxidase (also known as interleukin 4-induced gene-1, IL4I1) mRNA and protein in primary alveolar epithelial cells from immunocompetent mice infected intratracheally with *A. fumigatus* spores for 24 h [72]. Via immunofluorescence-mediated histopathology it was determined that IL4I1 was often co-localized with the presence, and in some cases even germination of, *A. fumigatus* conidia an observation which did not apply to tissues harvested from sham-infected mice. To assess the relevance of IL4I1 expression in human disease AEC II, in the vicinity of human aspergilloma lesions, were analysed revealing clear positivity for IL4I1 in ten patient samples. In contrast, tissue from the same lung but more distant from mycetoma was negative for IL4I1 signal. IL4I1 belongs to the family of L-amino acid oxidases (LAAO), the major enzymatic function of which is catalysis of LAAO deamination, predominantly phenylalanine. Reported as also being abundant in B-cells through STAT6 activation and in antigen presenting cells, such as dendritic cells and macrophages, this work by Seddigh et al. yields the first published data of primary AEC II responses to *A. fumigatus* challenge, demonstrates for the first time that IL4I1 is present in primary murine AEC II during *A. fumigatus* infection, and adds weight to an intriguing theory that AEC II function as an antigen presenting cell type. In phagocytes IL4I1 abundance has been shown to have a direct bactericidal activity suggesting a plausible role in host protection against *A. fumigatus* infection [73]. Considering these facts, and coupled with the role of IL4I1 in driving M2 macrophage polarization [73], expression of IL4I1 by alveolar epithelia cells in response to *A. fumigatus* could be an important driver of airway hypersensitivity, allergic T helper 2 responses, and fibrosis.

## 5. Secreted Effectors of Epithelial Damage

In humans, exposure to *A. fumigatus* can lead to the development of allergic lung disease, including allergic bronchopulmonary aspergillosis (ABPA), and severe asthma with fungal sensitization (SAFS). The secreted products of cultured *A. fumigatus* have been recently demonstrated to provoke remodelling of the respiratory epithelium in immunocompetent BALB/c mice [41]. Repeated intranasal dosing with culture filtrate, conducted over the course of five weeks led to airway hyperreactivity as measured by whole-body plethysmography, ELISA, and cell counts. Significant increases were seen in the expression of classical Th2 cytokines (IL-4, IL-5, IL-13) and serum IgE in lung homogenate compared to PBS-treated control and significant airway remodelling revealed by histopathological analysis [41]. Th2 cytokine and IgE levels in mice challenged with culture filtrates harvested from *A. fumigatus* mutants lacking the Aspf5 or Aspf13 proteases were indistinguishable from those from mice challenged with wild type culture filtrates. However, total inflammatory and white blood cell counts revealed a significant reduction in total BALF cell and blood neutrophil content following challenge with culture filtrate from Δ*aspf5* and Δ*aspf13* mutants compared to challenge with wild-type culture filtrate. Further, immunohistochemistry of serial wax lung sections from mice challenged with Δ*aspf5* and Δ*aspf13* filtrate showed significantly reduced peribronchiole eosinophil counts and bronchiole goblet cells, as well as reduced sub-epithelial collagen deposition in the lung suggesting a role for Aspf5 and 13 in airway remodelling during *A. fumigatus* infection [41]. The presence of Aspf13 (also known as Alp1, a 33 kDa serine protease) in sputum has recently been reported to correlate with asthma severity in atopic patients, having been repeatedly detected in sputum from asthmatic but not healthy patients [74].

Bhushan et al. recently showed in HBECs that *A. fumigatus* extracts suppress JAK-STAT signalling, potentially promoting Th2 bias [75]. In a follow-up study, using CXCL10 transcription and protein expression, and NF-κB and tyrosine-protein phosphatase non-receptor type II (PTPNII) protein expression as markers of Th1 bias, Homma et al. showed that *A. fumigatus* extracts suppressed Th1 bias in primary normal human bronchial epithelial cells [76]. siRNA-mediated knockdown of the protease-activated receptor-2 (PAR-2) or PTPNII suppressed CXCL10 expression, thereby implicating PAR-2 activation in suppression of the endogenous epithelial IFN response, a pathway which ordinarily functions to keep Th2 and Th17 responses in check. *A. fumigatus* extract and culture filtrate also suppressed the activation of epithelial signal transducer and activator of transcription 1 (STAT 1) which is a Th1 cytokine signature transcription factor [75].

Gliotoxin (Gt) is a major secondary metabolite and virulence factor produced by *A. fumigatus*. It is an epipolythiodioxopiperazine with a reactive disulfide bridge. At moderate levels, Gt is a powerful immunosuppressant in T cells, macrophages and neutrophils. At higher levels (>250 ng/mL) it induces apoptotic cell death (reviewed in [77]). Gt also exhibits strong anti-angiogenic activity, disrupting the formation of capillaries [78].

Gt exposure in vitro, of lung epithelial cells of all types, induces cytoskeletal collapse, cell peeling, and apoptosis [42,43,44,45] (Figure 2). The signalling pathways mediating the response of lung epithelial cells to Gt have been analysed in A549 alveolar cells, primary type II alveolar cells, and BEAS-2B bronchial epithelial cells in culture [42,44]. In A549 cells, sub-lethal Gt concentrations increased the internalization of *A. fumigatus* conidia approximately five-fold by stimulating PLD activity and actin reorganization. This might protect the fungus from immune cells and enable it to germinate into the surrounding tissue and capillaries. In primary type II alveolar and BEAS-2B cells, Gt activates the JNK pathway leading to apoptosis. JNK triple-phosphorylates the pro-apoptotic protein Bim, leading to pro-apoptotic Bak and Bax activation, mitochondrial membrane permeabilization and apoptosis.

Several studies have analysed the importance of Gt during in vivo infection, however, its effect on the integrity of the lung epithelium remains unclear. Gt is secreted by *A. fumigatus* during murine and human infection and can be detected in the lungs at bioactive levels [79,80]. Abolition of Gt biosynthesis by deletion of GliP, encoding the Gt peptide synthase, strongly reduces the cytotoxic activity of culture filtrates and results in attenuated virulence in infected non-neutropenic mice [81,82]. Attenuated virulence in this model was attributed to reduced apoptosis of neutrophils entering the lungs of mice infected by the Gt null mutant, resulting in improved host survival. Interestingly, in neutropenic mice, the *A. fumigatus* Gt null mutant was as virulent as the wild-type strain. This suggests that, in the absence of immune cells, when the fungus interacts only with lung epithelial cells, Gt apparently plays no active role in exacerbating the infection. This is surprising, as we know that in vitro Gt kills epithelial cells. Clearly, these results suggest that a more detailed microscopic analysis of the lung epithelial cell layer needs to be performed in neutropenic mice infected with wild-type and Gt null *A. fumigatus*.

## 6. Conclusions

Recent studies have delineated morphotype-specific epithelial responses to conidial and germinated forms of *A. fumigatus*. Given that germinated forms of *A. fumigatus* pose a major threat, both in vivo and in vitro, to epithelial integrity, and that epithelial cells can potently neutralise the vast majority of internalised spores [6], we, and others [72], hypothesise that epithelial cells, in collaboration with professional phagoctyes, provide an important, directly microbicidal, defence against *A. fumigatus* which can become dysfunctional in settings of respiratory disease or immune compromise. Within this context, epithelial entry would serve, in the healthy host, as a means to control everyday *A. fumigatus* exposure, and, in the diseased setting, as a critical gateway through which to elicit host entry and damage. Opportunities to study airway infection have been severely limited by the difficulties associated with isolating and culturing primary Type I and II cells. However, a stably transformed Type I cell line has been generated recently [83] and a move towards new proteomic and single-cell technologies will move the field on further still [84]. The experimental heterogeneity and intrinsic limitations of population-scale in vitro analysis leave several unanswered questions with regards to the mode of uptake of *A. fumigatus* by AECs and the relevance of this process in the context of mammalian disease. Multiple studies report spore internalisation by immortalised and/or primary AECs in in vitro infection systems and ex vivo organ culture models [6,7,8,9,10,11,12,13,14], but no compelling published evidence of in vivo spore internalisation by AECs is available. Several, currently insurmountable, technical challenges might be usefully circumvented by the use of computational approaches. For example, in support of a protective role for AEC-mediated activities, agent-based modelling of the human alveolus predicts that chemotactic signalling by AECs is required for the efficient recruitment of alveolar macrophages during early *A. fumigatus* infection [85] and low-dose exposure to *A. fumigatus* spores prompts fungal persistence in both in silico and murine models of pulmonary aspergillosis [85].

Studies of Aspergillus-epithelial interactions remain in their infancy and have not yet progressed well enough to meaningfully consider the important implications of the resident microbiota and/or biofilm formation. Co-infection studies, both in vitro and in vivo, are lacking and fungal hyphae are too noxious to in vitro-cultured epithelial cells to promote longevity of co-cultured AECs and fungal hyphae. Although the propensity to form biofilms is not hindered by co-culture with human bronchial epithelial cells [86], too few intact epithelial cells remain after 2–3 days of co-culture to promote the study of the host-pathogen interaction over a sustained period of time. Similarly, whilst it is known that co-habiting microbes, such as *Pseudomonas aeruginosa* can manipulate redox and iron homeostasis of *A. fumigatus* in in vitro co-culture [87], it is unknown how this might impact upon the outcome of host-pathogen interactions at the epithelial interface neither in healthy or diseased settings. Since physiologically-relevant studies of such aspects of disease demand whole animal studies performed in real time, it is likely that advances in imaging technology will provide the opportunity to surmount the technical hurdles still obscuring a full understanding of *Aspergillus*-host interactions with relevant inoculum sizes, host cell context, and resident microbiota.
